# Atomically thin resonant tunnel diodes built from synthetic van der Waals heterostructures

**DOI:** 10.1038/ncomms8311

**Published:** 2015-06-19

**Authors:** Yu-Chuan Lin, Ram Krishna Ghosh, Rafik Addou, Ning Lu, Sarah M. Eichfeld, Hui Zhu, Ming-Yang Li, Xin Peng, Moon J. Kim, Lain-Jong Li, Robert M. Wallace, Suman Datta, Joshua A. Robinson

**Affiliations:** 1 Department of Materials Science and Engineering and Center for 2-Dimensional and Layered Materials, The Pennsylvania State University, University Park, Pennsylvania 16802, USA; 2 Department of Electrical Engineering, The Pennsylvania State University, University Park, Pennsylvania 16802, USA; 3 Department of Materials Science and Engineering, The University of Texas at Dallas, Richardson, Texas 75080, USA; 4 Institute of Atomic and Molecular Sciences, Academia Sinica, Taipei 10617, Taiwan; 5 Physical Science and Engineering, King Abdullah University of Science and Technology, Thuwal 23955-6900, Saudi Arabia

## Abstract

Vertical integration of two-dimensional van der Waals materials is predicted to lead to novel electronic and optical properties not found in the constituent layers. Here, we present the direct synthesis of two unique, atomically thin, multi-junction heterostructures by combining graphene with the monolayer transition-metal dichalcogenides: molybdenum disulfide (MoS_2_), molybdenum diselenide (MoSe_2_) and tungsten diselenide (WSe_2_). The realization of MoS_2_–WSe_2_–graphene and WSe_2_–MoS_2_–graphene heterostructures leads to resonant tunnelling in an atomically thin stack with spectrally narrow, room temperature negative differential resistance characteristics.

## Abstract (web summary)

The family of two-dimensional materials is ever growing, but greater functionality can be realized by combining them together. Here, the authors report the direct synthesis of multijunction heterostructures made from graphene, tungsten diselenide and either molybdenum disulphide or molybdenum diselenide.

## Graphical abstract

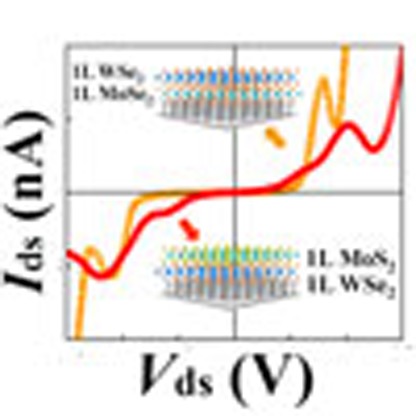

Resonant tunnelling of charge carriers between two spatially separated quantum states can lead to a unique current transport phenomenon known as negative differential resistance (NDR)^1^,^2^. This is a key feature for novel nanoelectronic circuits that utilize bistability and positive feedback, such as novel memories, multi-valued logic, inductor-free compact oscillators and many other not-yet-realized electronic applications^3^,^4^. However, realizing spectrally narrow NDR in a resonant tunnelling diode (RTD) at room temperature has been challenging due to carrier scattering related to interfacial imperfections, which are unavoidable in traditional semiconductor heterostructures synthesized using advanced epitaxial growth techniques^5^. Two-dimensional (2D) materials^6^,^7^, with no out-of-plane chemical bonding and pristine interfaces, presents an appealing alternative to traditional semiconductors, and could ultimately eliminate the interfacial imperfections that limit room temperature NDR performance to date. Since 2004 (ref. 6), the overwhelming majority of electronic transport and stacked in 2D materials has been reported using mechanically exfoliated flakes^8^. Recently, there has been a concerted effort to directly synthesize layered transition-metal dichalcogenides (TMDs), with powder vapourization^9^,^10^,^11^ synthesis paving the way for direct growth of atomically thin structures^9^,^10^,^11^,^12^,^13^,^14^. Beyond monolayer (ML) TMDs, van der Waals (vdW) heterostructures (heterogeneous stacks of dissimilar atomic layers) have been predicted to lead to novel electronic properties not found in their constituent layers^15^, where their realization has primarily come from mechanical exfoliation and stacking^16^,^17^,^18^,^19^. Manual stacking has provided experimental verification of electronic bandgap modulations and strong interlayer coupling^20^, but it can also lead to interface contamination^19^ that introduces additional scattering mechanisms and inhibits the NDR. Therefore, a synthetic route to achieve vdW heterostructures with pristine interfaces will be a critical step in the advancement of the field.

Here we present the direct synthesis of MoS_2_–WSe_2_–graphene and WSe_2_–MoSe_2_–graphene heterostructures employing a combination of oxide powder vapourization and metal-organic chemical vapour deposition (MOCVD). We not only demonstrate that these heterostructures exhibit the same interlayer electronic coupling found in mechanically exfoliated heterostructures^20^,^21^,^22^, but also show that they exhibit unique electronic transport properties not typically found in exfoliated structures. We discover that direct grown heterostructures exhibit resonant tunnelling of charge carriers, which leads to sharp NDR at room temperature. Importantly, we identify that the peak of the resonant tunnelling can be tuned by modifying the stacking order or layer composition, which will be a powerful tool towards engineering heterostructures for ultra-low power electronic devices.

## Results

### Formation of vertical vdW heterostructures

**Figure 1:**
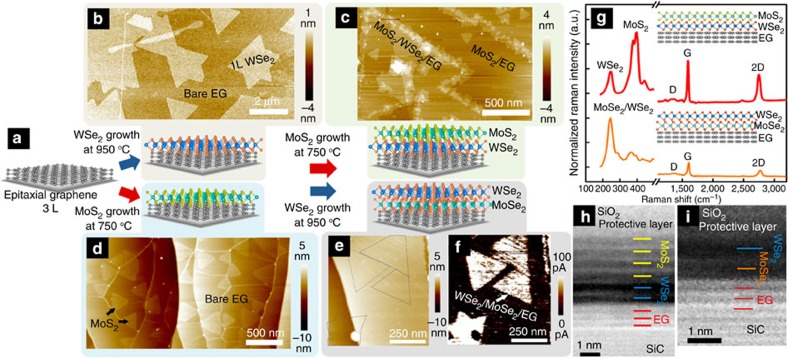
The formation of vdW heterostructures. MoS_2_–WSe_2_–EG vertical heterostructures begins with the synthesis of (**a**) 3L EG from SiC followed by (**b**) vapour transport or MOCVD of WSe_2_ and (**c**) vapour transport of MoS_2_. WSe_2_–MoSe_2_–EG heterostructures are similarly grown, except when (**d**) MoS_2_ is grown first on EG followed by (**e**) growth of the WSe_2_, a Se–S ion exchange occurs, leading to the formation of MoSe_2_ from the original MoS_2_ layer. The MoSe_2_ domains are difficult to topographically identify; however, (**f**) conductive AFM clearly delineates their location due to enhanced tunnelling at the heterostructures. Raman (**g**) indicates that preservation of the graphene has occurred during the synthesis process, and Scanning TEM (**h**,**i**) confirms that the stacked structures exhibit pristine interfaces, with no intermixing of Mo–W or S–Se after synthesis.

The heterostructure is achieved by sequentially growing two dissimilar TMD MLs on multilayer (three layers) epitaxial graphene (EG) (Fig. 1a)^23^. The individual TMD layers are grown *ex situ* via powder vapourization or MOCVD. Tungsten diselenide is synthesized using both routes: tungsten trioxide (WO_3_) and selenium (Se) powders for the powder vapourization route^24^, and tungsten hexacarbonyl (W(CO)_6_) and dimethylselenium ((CH_4_)_2_Se) for the MOCVD route^25^. Molybdenum disulfide is grown via vapourization of molybdenum trioxide (MoO_3_) and sulfur^10^. The heterostructure synthesis process is summarized in Fig. 1. The first TMD layer of the heterostructure, WSe_2_ or MoS_2_, is grown on tri-layer EG (Fig. 1a) at 950 °C and 750 °C for WSe_2_–EG (Fig. 1b) and MoS_2_–EG (Fig. 1c,d), respectively. Following this first TMD growth step, the surface coverage of the WSe_2_ or MoS_2_ on EG is typically >60%, with a lateral size of 2 μm and 300 nm for WSe_2_ and MoS_2_, respectively. Subsequently, the MoS_2_–WSe_2_–EG vertical heterostructure is created via a second *ex situ* growth of MoS_2_ on WSe_2_–EG at 750 °C (Fig. 1c). Similar to our previous work^26^, we find that wrinkles in the graphene as well as defects and edges within the WSe_2_ promote vertical growth of the MoS_2_, and ML MoS_2_/WSe_2_ is primarily achieved in pristine regions of WSe_2_ (Fig. 1c and Supplementary Fig. 1a)^26^. The formation of the WSe_2_–MoSe_2_–EG heterojunction occurs during growth of WSe_2_ on MoS_2_. During the synthesis, a selenium–sulfur ion exchange occurs when the MoS_2_ is exposed to the selenium vapour just prior to the growth of WSe_2_ at 950 °C for 45 min^27^. Standard topographic characterization via atomic force microscopy (AFM) cannot clearly identify the location of the heterostructures (Fig. 1f); however, conductive AFM (CAFM) with platinum (Pt) tip^28^ provides a means to map the WSe_2_–MoSe_2_–EG junctions and adjacent WSe_2_–EG regions due to a difference in heterostructure conductivity (Fig. 1f and Supplementary Fig. 1c,d).

Raman spectroscopy and transmission electron microscopy (TEM) confirm the formation of crystalline, vertical heterostructures (Fig. 1g–i and Supplementary Figs 1–3). A large fraction of the EG remains nearly defect free following the sequence of TMD growths; however, there are regions of increased defectiveness due to either partial passivation of the graphene/SiC buffer layer^23^ or formation of thick TMD layers^26^. Raman spectroscopy (see Supplementary Fig. 3) also confirms presence of significant fractions of ML WSe_2_ (*E*_2g_/*A*_1g_ at 250 cm^−1^ and 2LA at 263 cm^−1^)^24^ and MoS_2_ (*E*_2g_ at 383 cm^−1^ and *A*_1g_ at 404 cm^−1^)^26^, as well as ML MoSe_2_ (*A*_1g_ at 240 cm^−1^ and *E*^1^_2g_ at 284 cm^−1^)^27^. X-ray photoelectron spectroscopy (see Supplementary Fig. 4 and Supplementary Table 1) also corroborates the absence of any interaction between the two TMDs or graphene, and indicates that the MoS_2_ exhibits an n-type behaviour, while the WSe_2_ layer shows a p-type behaviour. Scanning TEM (Fig. 1h,i) also verifies the heterostructure is not a manifestation of the alloying of the constituent TMDs, but indeed are unique layers with pristine interfaces with atomic precision. In the case of MoS_2_–WSe_2_–EG, we have focused on a multilayer region of MoS_2_–WSe_2_ to ensure pristine layer formation beyond the ML structure (see Supplementary Fig. 2); however, all electrical characterization presented later is on ML heterostructures. The clean interface between MLs can be observed easily using high resolution scanning TEM. The WSe_2_–MoSe_2_–EG ordering is confirmed by comparing the intensity with that of bilayer WSe_2_–EG due to the similar atomic number between W and Mo atom (see Supplementary Fig. 2). Unlike vertical heterostructures based on a single chalcogen (that is, MoS_2_/WS_2_)^29^, the ordered layering does not occur when we attempt to grow a vertical structure based on heterogeneous layers where *M*_1_≠*M*_2_ and *X*_1_≠*X*_2_ (*M*=Mo, W; *X*=S, Se) on ‘3D' substrates such as sapphire or SiO_2_ (see Supplementary Fig. 5). Instead, all attempts to grow such a structure results in alloying or lateral heterostructures of the layers. Therefore, we hypothesize that EG plays a critical role in the formation of atomically precise vdW heterostructures, where *M*_1_≠*M*_2_ and *X*_1_≠*X*_2_ by providing an atomically smooth surface that is free of dangling bonds, enabling mobility on the surface for TMD layer growth. Sapphire and SiO_2_ surfaces exhibit high surface roughness, dangling bonds, and are therefore more likely to impede surface diffusion, which catalyzes the alloying process.

### Interlayer coupling

**Figure 2:**
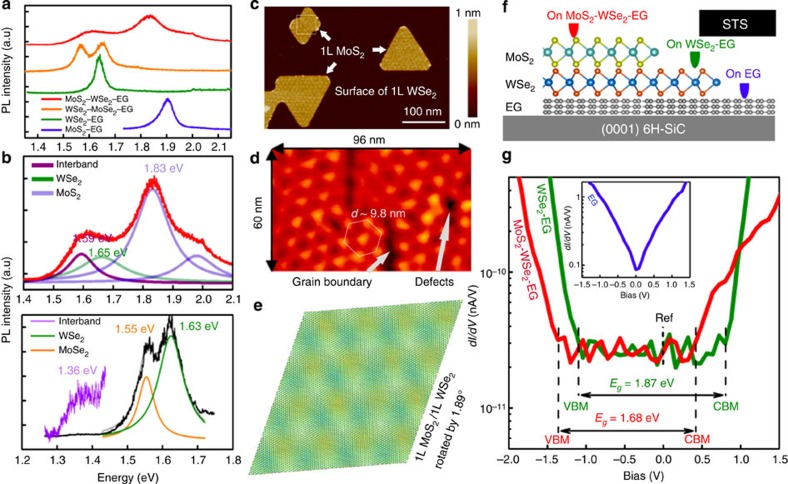
Coupling in 2D vertical heterostructures. (**a**) The PL properties of MoS_2_–WSe_2_–EG and WSe_2_–MoSe_2_–EG reveal significant interlayer coupling, where the (**b**) MoS_2_–WSe_2_–EG and WSe_2_–MoSe_2_–EG exhibit the intrinsic PL peaks corresponding to MoS_2_, MoSe_2_ and WSe_2_, and also exhibit interband PL peaks at 1.59 and 1.36 eV, where the excitation wavelength (*λ*) is 488 nm and 633 nm for MoS_2_–WSe_2_–EG and WSe_2_–MoSe_2_–EG, respectively. (**c**) The moiré patterns acquired via AFM in MoS_2_ on WSe_2_ indicates an alignment of nearly either 0° or 180° between the top and bottom layer, and (**d**) STM confirms the moiré pattern with a lattice constant equal to (9.8±0.4) nm. This structure can be reproduced theoretically (**e**) when the misorientation angle between these layers is ∼1.9°. The continuity of the Moiré pattern is interrupted by the formation of a grain boundary and point defects, as indicated in the STM image. (**f**,**g**) STS on MoS_2_–WSe_2_–EG, WSe_2_–EG and EG (**g**, inset) provide evidence that the bandgap of the double junction heterostructure (MoS_2_–WSe_2_–EG) is smaller than that of the single-junction (WSe_2_–EG) heterostructure. The positions of conduction band minimum (CBM), valence band maximum (VBM), and quasi-particle bandgap *E*_g_ of WSe_2_ on EG and bilayer on EG are marked.

ML-semiconducting TMDs exhibit a direct optical bandgap (*E*_opt_) (MoS_2_ at 1.8∼1.9 eV, MoSe_2_ at 1.55 eV, and WSe_2_ at 1.6∼1.65 eV)^30^; therefore, photoluminescence (PL) spectroscopy (Fig. 2a,b) can provide evidence of electronic coupling between the layers. In addition to the typical PL peaks from the direct bandgap transition within the individual layers, the PL spectra of the heterostructures exhibit the presence of interlayer excitons at 1.59 eV for MoS_2_–WSe_2_–EG and 1.36 eV for WSe_2_–MoSe_2_–EG (see Fig. 2a,b). In this case, the MoS_2_–WSe_2_ and WSe_2_–MoSe_2_ junctions exhibit type II band alignment^15^,^20^,^21^,^31^, where electrons in the WSe_2_ conduction band transfer to the conduction band of MoS_2_ (MoSe_2_) and the excited holes in MoS_2_ (MoSe_2_) valence band transfer to the valence band of WSe_2_. Consistent with manually stacked heterojunctions^20^,^21^, the position of the PL peak is due to interlayer exciton recombination, which confirms the electronic coupling at the heterojunction between the two ML TMDs.

Additional evidence of coupling comes from the topographical information of the heterostructures. Similar to graphene–hBN heterostructures^32^, Moiré patterns of MoS_2_–WSe_2_ are observed in tapping-mode AFM, which are qualitatively consistent with rotation angles of ∼0 or 180° between MoS_2_ and WSe_2_. Furthermore, scanning tunnelling microscopy/spectroscopy (Fig. 2d) confirms the presence of a Moiré pattern produced by the misorientation of MoS_2_ relative to the underlying WSe_2_ layer. The lattice constant of the Moiré pattern is 9.8±0.4 nm, which corresponds to a misorientation angle of ∼1.9°. Modelling the heterostructure with this misorientation produces a consistent Moiré pattern, with a slightly smaller lattice constant of 9.6 nm (Fig. 2e). While the mechanical stacking technique leads to a variety of rotation angles between layers^20^, the direct growth of vdW layers using our approach appears to have a strict rotational alignment, which may be critical for achieving optimal coupling between the layers^33^,^34^.

Scanning tunnelling spectroscopy further provides evidence that the quasi-particle bandgap of MoS_2_–WSe_2_–EG is significantly smaller than its WSe_2_–EG counterpart (Fig. 2f,g, and Supplementary Fig. 6). Based on STS, we infer that, for WSe_2_–EG, the conduction band minimum is at a sample bias of +0.71±0.08 V and the valence band maximum is at −1.11±0.02 V (green curve in Fig. 2g). This indicates that the quasi-particle bandgap (*E*_g_) of WSe_2_ is 1.83±0.05 eV, which is higher than *E*_opt_ (1.63 eV) due to the large excitonic binding energy in 2D TMDs^14^,^22^,^31^,^35^. On the other hand, MoS_2_–WSe_2_–EG exhibits a conduction band minimum at +0.34±0.03 V and valence band maximum at −1.31±0.03 V, indicating a quasi-particle interlayer *E*_g_ of 1.65 eV±0.02 V, which is slightly larger than its interlayer *E*_opt_ at 1.59 eV (Fig. 2b) but smaller than the *E*_opt_ in 1L MoS_2_–EG^22^,^31^. Mapping the tunnel current density of WSe_2_–EG and WSe_2_–MoSe_2_–EG heterostructures via CAFM^28^,^36^ (Fig. 1f and Supplementary Fig. 1) provides strong evidence that tunnelling is much more readily achieved in WSe_2_–MoSe_2_–EG at a tip bias of 1.5 V, indicating a smaller, resonance tunnelling or both may be occurring. Finally, we note that defects, such as grain boundaries and vacancies disrupt the continuity of the Moiré pattern, further emphasizing that imperfections in layers or the interface will significantly impact the electronic behaviour of vdW heterostructures (Fig. 2d).

### Vertical transport

**Figure 3:**
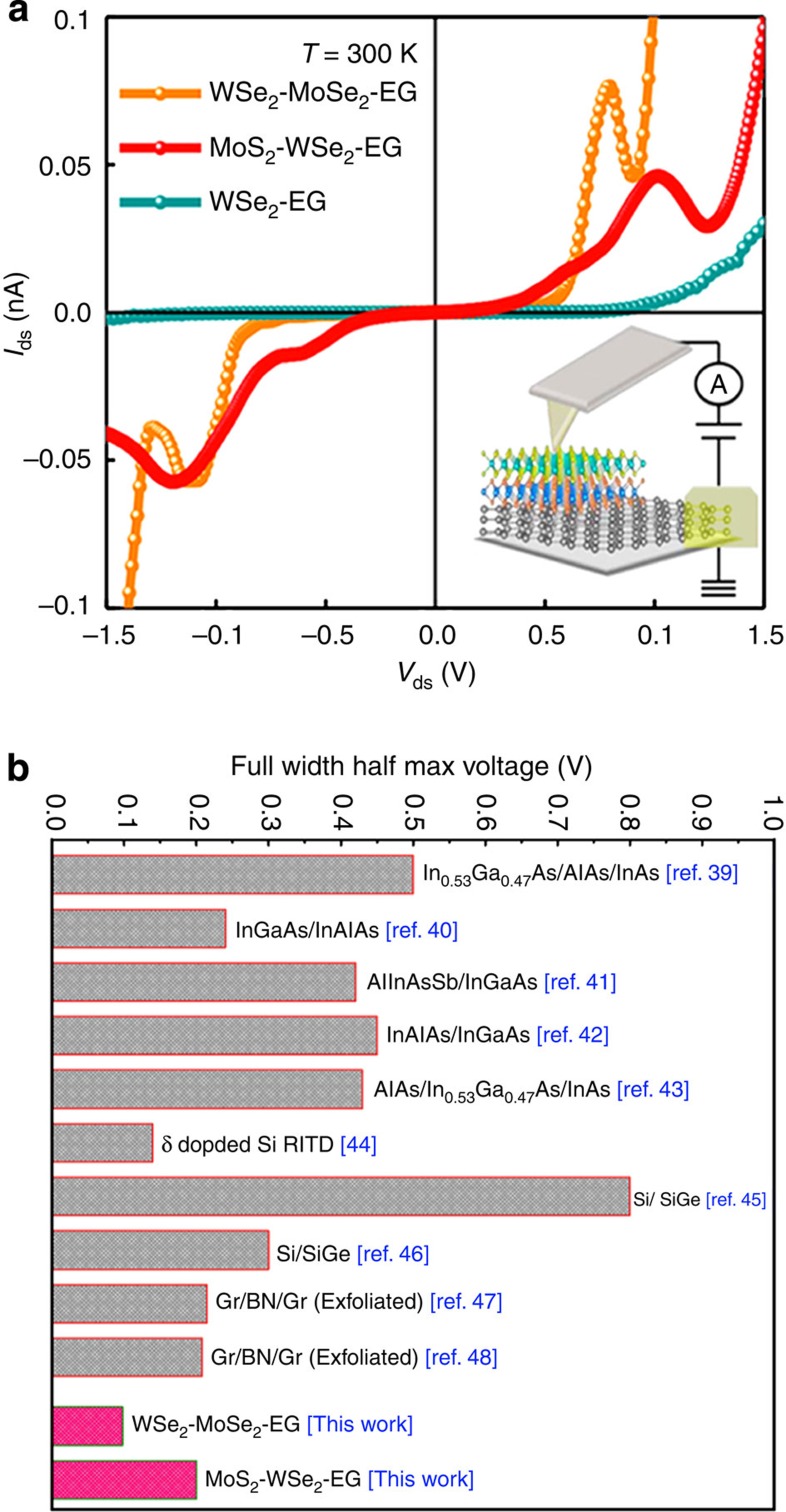
Resonant tunnelling and negative differential resistance in atomically thin layers. (**a**) Experimental *I–V* traces for different combination of dichalcogenide–graphene interfaces demonstrating NDR. The inset shows schematic of the experimental set-up for the *I–V* measurement in this layered system. (**b**) Comparison of full width at half maximum voltage of the NDR from this work with other reported results in room temperature^40^,^41^,^42^,^43^,^44^,^45^,^46^,^47^,^48^,^49^.

Room temperature current–voltage measurements through the heterostructure (carried out via CAFM) do not exhibit the traditional p–n junction diode-like transport found in mechanically exfoliated dichalcogenide structures or direct grown single-junction (that is, WSe_2_–EG) structures^20^,^26^,^37^. Instead, we find that, following a ‘soft' turn-on, the current exhibits a peak at a certain bias voltage (*V*_peak_=+1.1 and +0.7 for MoS_2_–WSe_2_–EG and WSe_2_–MoSe_2_–EG, respectively), then decreases to a minimum before undergoing a ‘hard' turn-on with exponential current increase (Fig. 3a). The peak-to-valley current ratio is 1.9 for MoS_2_–WSe_2_–EG and 2.2 for WSe_2_–MoSe_2_–EG (Fig. 3a and Supplementary Fig. 7, and Supplementary Table 2), which is comparable to traditional RTDs^1^,^2^,^3^,^4^,^5^.

## Discussion

We have demonstrated the direct synthesis of unique multi-junction heterostructures based on graphene (EG on SiC), MoS_2_, MoSe_2_ and WSe_2_ that yields pristine interlayer gaps and leads to the first demonstration of resonant tunnelling in a atomically thin synthetic stack with the spectrally narrowest room temperature NDR characteristics. Importantly, this work indicates that NDR at room temperature only occurs in TMD heterostructures with truly pristine interfaces, which has been recently corroborated with manually stacked heterostructures where NDR is only evident at liquid nitrogen temperatures^20^,^38^,^39^. This is due to resonant tunnelling being highly sensitive to interfacial perturbations such as defects or ‘residue' from the transfer process, emphasizing the importance of direct synthesis of multi-junction TMD heterostructures for vertical quantum electronics applications. Interestingly, the room temperature full width at half maximum of the NDR in this work is more spectrally narrow than their ‘3D' semiconductor counterparts (silicon, germanium, III–V) and manually stacked graphene–boron nitride–graphene (Gr–hBN–Gr) heterostructures (Fig. 3b and Supplementary Note 7)^40^,^41^,^42^,^43^,^44^,^45^,^46^,^47^,^48^,^49^. This suggests that the interface of the directly grown vdW heterostructures is superior to that of many previously reported RTD structures.

## Methods

### EG synthesized from 6H-SiC

Graphene is synthesized on 1 cm^2^ squares of 6H-SiC (0001) in a graphite crucible^23^. The 6H-SiC substrate was annealed in H_2_ at 1,500 °C for 10 min to clean substrate surface prior to graphene growth. At this stage the chamber pressure is 700 torr under a H_2_ (50 s.c.c.m)/Ar (450 s.c.c.m) flow. After H_2_ annealing, the system temperature cooled to 850 °C and pumped/purged with ultra-high pure N_2_ at least six times to remove H_2_ gas. Subsequently the chamber is filled in Ar gas (500 s.c.c.m.) to 200 torr. The chamber was then heated up to 1,725 °C at 100 °C min^−1^ and dwelled at this temperature for 20 min to grow three layers of graphene within the terraces of substrates via sublimation of silicon on the silicon side of 6H-SiC (0001). The system cooled down naturally to room temperature after the growth.

### MoS_2_–WSe_2_–EG synthesis

WSe_2_ can be grown on EG either via vapour phase reaction of WO_3_ and Se powders or via MOCVD^24^,^25^. The vapour phase reaction utilizes the vapourization of WO_3_ powders in a ceramic boat placed at the centre of 1′′ horizontal hot wall tube reactor with a flow of H_2_ (10 s.c.c.m.)/Ar (90 s.c.c.m.). The EG substrates for WSe_2_ growth were placed at the downstream side of the tube and heated to 925 °C at 25 °C min^−1^. Samples were held at 925 °C for 15 min and then cooled naturally to room temperature. The total pressure throughout the reaction is held at 6 torr. Utilizing MOCVD, WSe_2_ was synthesized in a vertical cold wall reactor using W(CO)_6_ and (CH_4_)_2_Se precursors. The metallic organic precursors were transported into the reactor by carrier gas of 100% H_2_ via a bubbler manifold that allows for controlling each precursor concentration independently. The (CH_4_)_2_Se and W(CO)_6_ were held at temperatures of 22 °C and 25 °C, respectively, and a pressure of 760 torr. The Se to W ratio was fixed at 20,000. The MOCVD growth of WSe_2_ took place at 800 to 850 °C with a total pressure of 700 torr. The growth time varied between 15 and 30 min. After the completion of WSe_2_–EG synthesis, the *ex situ* MoS_2_ growth via the vapour phase reaction using MoO_3_ and S powders was carried out in a horizontal hot wall tube reactor at 700 torr. During the MoS_2_ growth, MoO_3_ powder in a ceramic boat placed at the centre of heating zone were heated at 750 °C for 10 min. After the MoS_2_ growth, the reactor cooled down to room temperature naturally.

### WSe_2_–MoSe_2_–EG synthesis

In this case, the processes are similar, but steps reversed. The MoS_2_ is grown first, followed by an *ex situ* WSe_2_ growth via the vapour phase reaction of WO_3_ and Se. A Se–S ion exchange occurs in the MoS_2_ converting the MoS_2_ into MoSe_2_^27^. Subsequently, the WSe_2_ layers grow on MoSe_2_–EG as the hotzone is held at 950 °C for 45 min, resulting in WSe_2_–MoSe_2_–EG heterostructures.

### Sample characterization

The details of characterizations performed on the heterostructures can be found in the Supplementary Note 1.

## Additional information

**How to cite this article:** Lin, Y-C. *et al.* Atomically thin resonant tunnel diodes built from synthetic van der Waals heterostructures. *Nat. Commun.* 6:7311 doi: 10.1038/ncomms8311 (2015).

## Supplementary Material

Supplementary Information Supplementary Figures 1-7, Supplementary Tables 1-2, Supplementary Notes 1-7 and Supplementary References.
