# Supplementary material for: Atomically thin resonant tunnel diodes built from synthetic van der Waals heterostructures
Source: Nat Commun. 2015 Jun 19;6:7311. doi: 10.1038/ncomms8311 (PMC4557306; doi:10.1038/ncomms8311)
Supplement: Supplementary Information — Supplementary Figures 1-7, Supplementary Tables 1-2, Supplementary Notes 1-7 and Supplementary References. [file ncomms8311-s1.pdf]

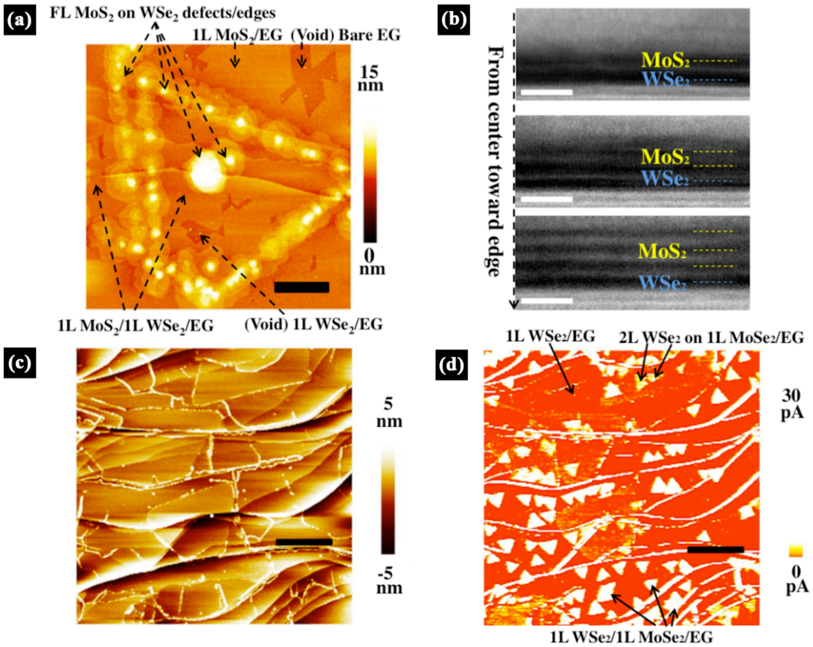

Supplementary Figure 1: MoS_2 crystals on WSe_2-EG and EG and WSe_2 crystals on $MoSe_2$-EG and EG. (a) The MoS_2 crystals cover both of EG and WSe_2/EG after the CVD growth (Scar bar: 400 nm) (b) shows TEM profiles across the MoS_2-WSe_2-EG: Most of area in WSe_2 has MoS_2 in monolayer (ML) thickness while the area surrounding the edge has multilayered MoS_2 potnetially due to higher reactivity around edges and defects, as shown in (a). (Scale bar in all the TEM image: 2 nm). (c,d) WSe_2 can keep growing on ML WSe_2-ML $MoSe_2$-EG and evatually formed bilayer and above WSe_2 on $MoSe_2$-EG (c), which reduces the amplitude of tunnel current on the trilayered junciton. (Scar bar in (c,d): 1 μm; The V_{bias} in (d) is + 0.8 V)

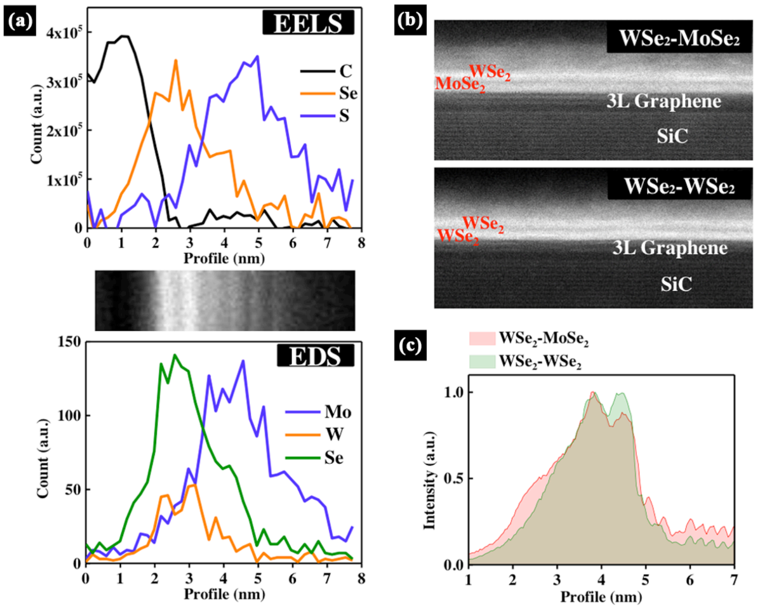

Supplementary Figure 2: Electron energy loss spectroscopy (EELS) and Electron diffraction spectroscopy (EDS). (a) The EELS and EDS cross-profiles in HAADF HRTEM of double junctions elemental distributions of MoS_2, WSe_2, and graphene; and indicates no alloys in each TMD layer. (b,c) The WSe_2-$MoSe_2$ area has significant contrast lost comparing to WSe_2-WSe_2 area (top and bottom HAADF), due to $MoSe_2$ layer.

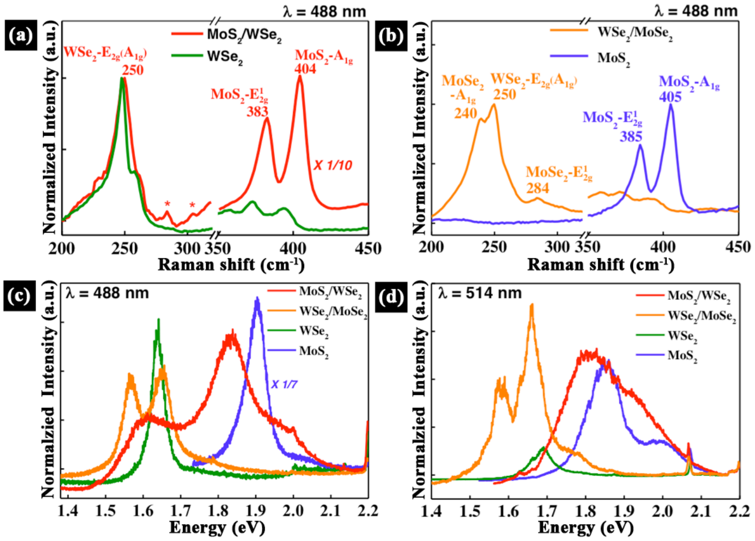

Supplementary Figure 3: The Raman PL spectra of heterostructures. (a) The spectrums clearly displays distinct features from MoS_2/WSe_2, (b) and $WSe_2/MoSe_2$ and indicate no alloy-like features. The asterisks indicate the signatures of their strong couplings and can also be found in mechanically stacked MoS_2/WSe_2.[1] (c) and (d) show the PL of heterostructures with intensity normalized to the Raman feature of SiC substrate with 488 and 514 nm excited laser, respectively. The peaks features in terms of signal intensity and peak shapes have been significant modulated versus the peaks of MoS_2/EG and WSe_2/EG.

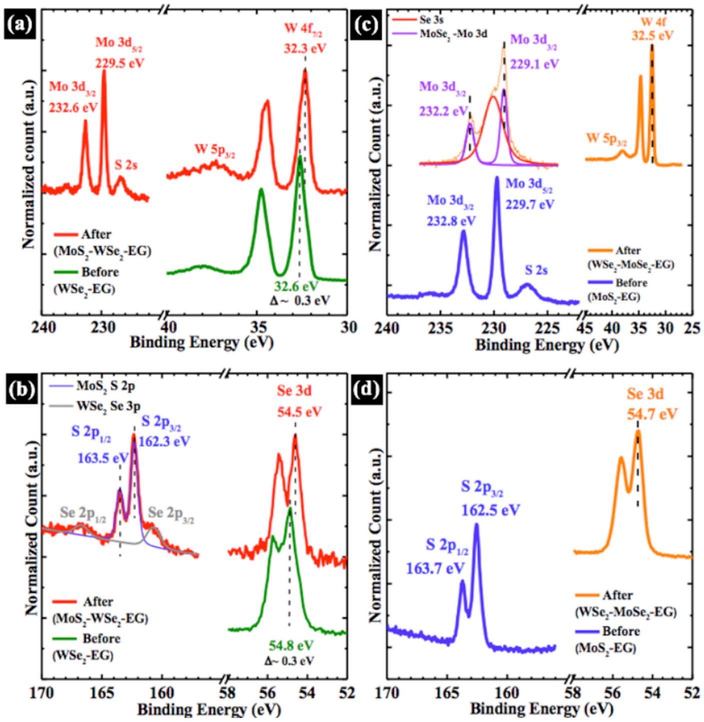

Supplementary Figure 4: XPS core shell analysis of (a,b) MoS_2/WSe_2/EG and (c,d) $WSe_2/MoSe_2$/EG heterostructures. The photoemission indicates the absence of any interaction between the two TMDs or detectable TM-carbide formation with the underlying graphene/SiC. All oxides (W-O, Mo-O, Se-O, and S-O) are below the detection limit. In (c) the S 2*s* intensity associated with MoS_2/EG is also below the limit of detection after the WSe_2 growth, corresponding to the complete selenization of the MoS_2. Comparison of W 4f and Se 3d core shell doublet from WSe_2/EG and MoS_2/WSe_2/EG (a,b) showing an energy shift of ~0.3 eV, indicating $MoSe_2$ withdraws the negative charge to the bottom WSe_2.[2,3]

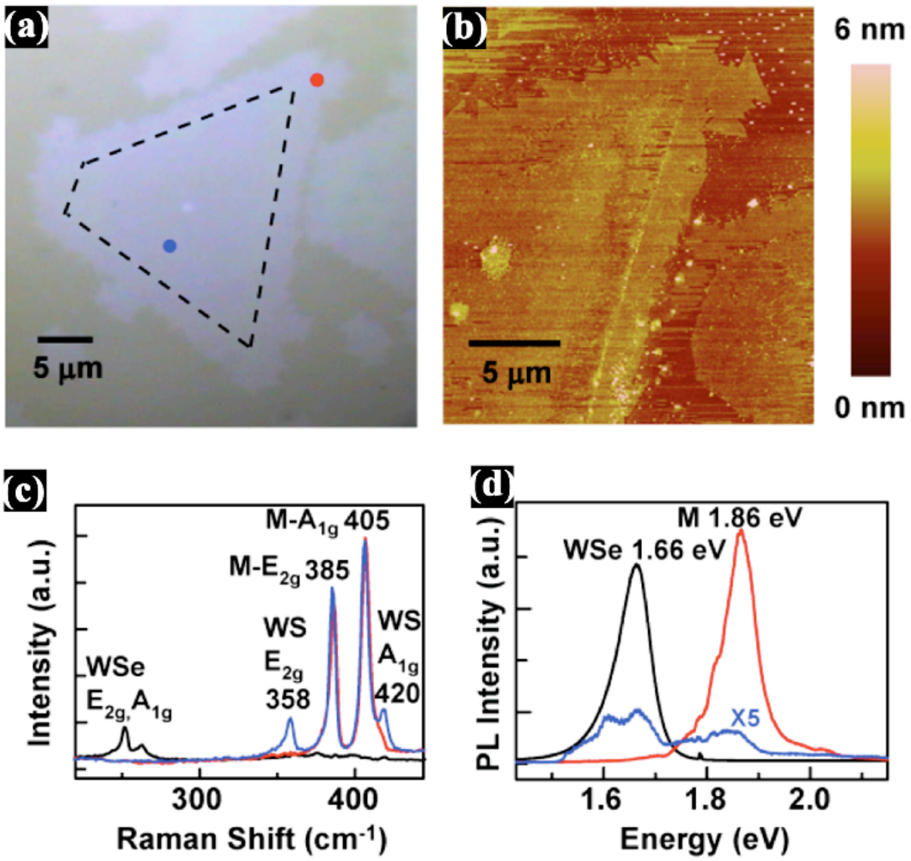

Supplementary Figure 5: The growth of vertical heterostructures on sapphire ends up with 2D alloys. (a) shows the optical micrograph after CVD process, and the boundary of per-growth WSe_2 part is located with black dashed line. In some case, we observed that MoS_2 is growth from the edge instead on the top of the WSe_2, which is clearly shown in (a). The AFM image of (b) confirms that the MoS_2 grows from the edge. Some structural damages on per-growth WSe_2 are found in (b). (c,d) show the Raman and PL spectrum of the WSe_2 before (black) and after (blue) MoS_2 synthesis compared to bare MoS_2 (red), the examined positions are indicated in (a).

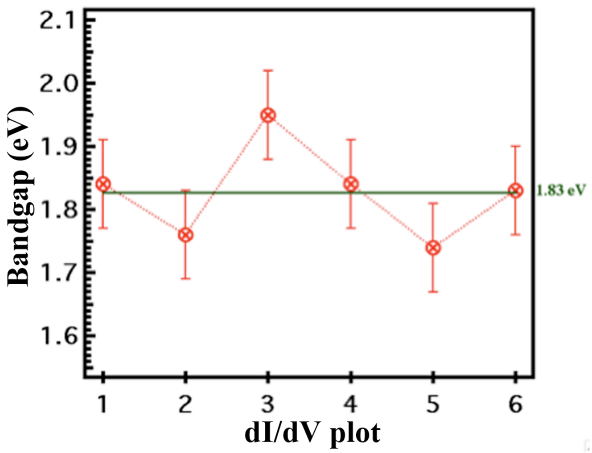

Supplementary Figure 6: WSe_2 band gap extrapolation. The value was extrapolated from six dI/dV curves.

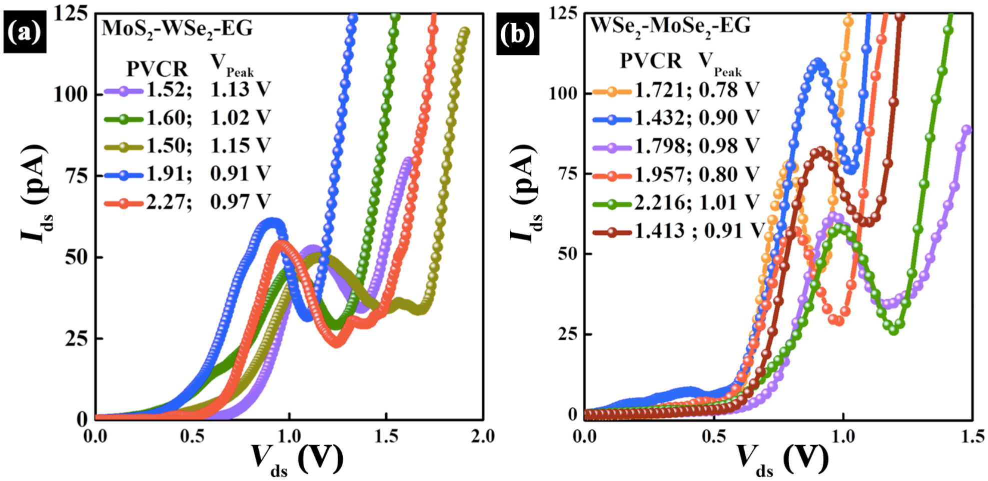

Supplementary Figure 7: Additional I-V plots. The additional NDR curves from MoS_2-WSe_2-EG and WSe_2-$MoSe_2$-EG heterostructures are plotted in (a) and (b), respectively.

Supplementary Table 1: XPS core level position measured for different interfaces.

	**C *1s*** *	**W $4f_{7/2}$**	**Se $3d_{5/2}$**	**Mo $3d_{5/2}$**	**S $2p_{3/2}$**
**EG**	**284.1**	**-**	**-**	**-**	**-**
**WSe_2/EG**	**284.4**	**32.6**	**54.8**	**-**	**-**
**MoS_2/EG**	**284.4**	**-**	**-**	**229.7**	**162.5**
**MoS_2/WSe_2/EG**	**284.3**	**32.3**	**54.5**	**229.5**	**162.3**
**WSe_2/$MoSe_2$/EG**	**284.4**	**32.5**	**54.7**	**229.1**	**-**
**Bulk samples**					
**n-MoS_2[4]**	**-**	**-**	**-**	**229.9**	**162.7**
**p-MoS_2[4]**	**-**	**-**	**-**	**229.1**	**161.9**
**p-WSe_2[5]**	**-**	**32.7**	**54.9**	**-**	**-**

* The C 1s peak was convoluted to 3 components (carbide + graphene + buffer layer), the table shows only the C 1s peak from graphene.

Supplementary Table 2: The comparison with other reported NDR.

System	V_{peak} (V)	J_{peak} (μA/μm^2)	J_{valley} (μA/μm^2)	PVCR
MoS_2/WSe_2/EG (This work)	0.8 to 1.1	0.15	0.075	1.5 to 2.3 (T=300K)
$WSe_2/MoSe_2$/EG (This work)	0.7 to 1.1	0.12	0.060	1.4 to 2.2 (T=300K)
Gr/BN/Gr [6]	0.8	0.22	0.13	1.7 (T=300K, no gating)
3-layered MoS_2 [7]	0.51	3	2.7	1.1 (T=60K, no gating)
Si/SiGe [8]	0.22	3	0.83	3.6 (T=300K)

Supplementary Note 1 Characterization Instrumentation

The as-grown heterostructures are characterized using Raman spectroscopy, atomic force microscopy/conductive atomic force microscopy (AFM/CAFM), X-ray photoelectron spectroscopy (XPS), and transmission electron microscopy (TEM). A WITec CRM200 Confocal Raman microscope with 488/514/633 nm wavelength lasers was utilized for structural characterization. A BRUKER Dimension with a scan rate of 0.5 Hz was utilized for the topography image during the AFM measurement. Conductive AFM (CAFM) measurement was performed in PeakForce TUNA mode with platinum (Pt) AFM tip. The applied voltage from tips to sample was increased from 0 to 2 V. The optimized loading force of the AFM tip and sensitivity was nominally 5 nN and 20 pA/V, respectively, for the I-V measurements carried out on the novel junctions. All the AFM/CAFM measurements in BRUKER Dimension were at room temperature and in ambient. TEM cross-sectional samples were made via utilizing a NanoLab dual-beam FIB/SEM system. Protective layers of SiO_2 and Pt were deposited to protect the interesting region during focused ion beam milling. TEM imaging was performed using a JEOL 2100F operated at 200 kV. For surface analysis, the sample was loaded into an ultra-high vacuum (UHV) with a base pressure lower than 2×10^{-10} mbar. The WSe_2/EG sample was then imaged using an Omicron variable temperature scanning tunneling microscope (STM) without any thermal treatment. The STM images were obtained at room temperature and in the constant-current mode, with an etched tungsten tip. The same system is equipped with a monochromatic Al-Kα source (E =1486.7 eV) and an Omicron Argus detector operating with pass energy of 15 eV. The spot size used during the acquisition is equal to 0.5 mm. Core-level spectra taken with 15 sweeps are analyzed with the spectral analysis software analyzer. (See: http://rdataa.com/aanalyzer).

Supplementary Note 2 Heterostructure Synthesis on Epitaxial Graphene Substrates.

Molybdenum disulfide synthesis on WSe_2/EG (Supplementary Figure 1a), often results in multilayer growth along edges and defects in WSe_2 due to higher reactivity at these sites. Cross-sectional HRTEM images of the MoS_2/WSe_2 heterostructure (Supplementary Figure 1b) scanned from center to edge of the heterostructures verify the increase in top layer thickness. The WSe_2/$MoSe_2$/EG heterostructures cannot be identified in AFM morphology without the assistance of CAFM (Supplementary Figure 1c,d). Bilayer WSe_2 can also form on $MoSe_2$/EG. The positions of stacking trilayer are visualized in CAFM under V_{bias} due to different electrical properties between WSe_2/EG and WSe_2/$MoSe_2$/EG (Supplementary Figure 1d). Electron energy loss spectra (EELS) and energy dispersive x-ray spectroscopy (EDS) data (Supplementary Figure 2a,b) verifies the heterostructures do not contain alloy but are instead unique layers with pristine interfaces. In the case of MoS_2-WSe_2-EG, a multilayer region of MoS_2-WSe_2 was focused to ensure pristine layer formation beyond the monolayer (ML) structure, however, all electrical characterization presented later is on ML heterostructures. The WSe_2-$MoSe_2$-EG ordering is confirmed by comparing the intensity with that of bilayer-WSe_2-EG due to the similar atomic number between W and Mo atom (Supplementary Figure 2c).

Supplementary Note 3 Raman, PL, and XPS of the Heterostructures.

Raman spectroscopy (Supplementary Figure 3a,b) confirms presence of significant fractions of ML WSe_2 (E_{2g} /A_{1g} at 250 cm^{-1} and 2LA at 263 cm^{-1})[9] and MoS_2 (E_{2g} at 383 cm^{-1} and A_{1g} at 404 cm^{-1}),[10] as well as ML $MoSe_2$ (A_{1g} at 240 cm^{-1} and E^{1}_{2g} at 284 cm^{-1}) and indicates the absence of alloying. The spectroscopic signatures due to the interlayer coupling of MoS_2/WSe_2 are located at 285 cm^{-1} (E'' for MoS_2)[1] and 309 cm^{-1} (out-of-plane mode A^{2}_{1g} for WSe_2)[1] (marked with asterisks in Supplementary Figure 3a). The normalized PL (Supplementary Figure 3c,d) under two different excited wavelengths also provide evidence that no alloying has occurred.

Prior to the TMD growth, XPS analysis was performed on epitaxial graphene (EG) synthetized on 6H-SiC(0001).[10] The C *1s* core binding energy levels were determined to be 284.1 eV and identical to the binding energy measured on a reference sample of HOPG. The core level spectra obtained after the growth of MoS_2 or WS_2 on EG are presented in Supplementary Figure 4 and compared in Supplementary Table 1. The C *1s* shifts by 0.3 eV to a higher binding energy after the formation of the first interface (MoS_2/EG or WSe_2/EG) indicating p-doped graphene. The C *1s* position remains the same after formation of the second interface in the trilayer heterostructure (MoS_2/WSe_2/EG or WSe_2/$MoSe_2$/EG).

For comparison, a n-type MoS_2 bulk crystal indicates that the core level of Mo $3d_{5/2}$ (S $2p_{3/2}$) is located at 229.9 eV (162.7 eV),[4] The W $4f_{7/2}$ (Se $3d_{5/2}$) core level measured on a p-type WSe_2 crystal is located at 32.4 eV (54.9 eV). This suggests that the MoS_2 film exhibits a n-type conductivity and the WSe_2 layer shows a p-type behavior. We explain the difference between the n-WSe_2 (STS) vs. p-WSe_2 (XPS) by the difference in the local measurement for STS ($\leq$ 1nm^2) vs. the surface sampling from the 0.5 mm diameter spot size used during the XPS acquisition. The

photoemission also indicates the absence of any interaction between the two transition metal dichalcogenides or carbide formation with the underlying substrate. Mo-O bond formation is the only oxide detected at 236.7 eV (Mo $3d_{3/2}$). W-O bond formation, if present, is below the limit of detection as the W $4f$ oxidized state overlaps with the broad W $5p$ peak located at 37.6 eV. The formation of WO_3 oxide is frequent occurrence during the growth, with a peak position of W $4f_{5/2}$ at 37.5 eV.[11] Moreover, the XPS analysis shows the absence of any detectable Se-O and S-O oxides. Even the Mo-O bond is below the limit of detection after a thermal treatment in UHV at 250 °C. Supplementary Table 1 provides the core level energies of WSe_2 and MoS_2 interfacing with graphene, which nearly the same position as the bulk samples p-WSe_2 and n-MoS_2. Interestingly, the doping level for WSe_2 in combination with graphene and MoS_2, is different in comparison to only WSe_2/graphene or WSe_2/$MoSe_2$. Also, MoS_2 is more n-type in a MoS_2/graphene than MoS_2/WSe_2.

Supplementary Note 4 Heterostructure Synthesis on Traditional Substrates.

Following the same processes described in the main text, we attempted to grow a vertical WSe_2/MoS_2 hetero-junction on a sapphire substrate. The detailed growth processes are described in the Method section of the main text. Supplementary Figure 4a shows an optical micrograph after the CVD process, where the pre-growth WSe_2 is marked with black dashed line. In some cases, we observed that MoS_2 grew from the edge rather than on top of the WSe_2, which is clearly shown in Supplementary Figure 5a. The AFM image of Supplementary Figure 5b confirms that the MoS_2 grows from the edge. We also find that there might have been some structural damage on pre-growth WSe_2 as found in Supplementary Figure 5b. Supplementary Figure 5c,d show the Raman and PL spectrum of the WSe_2 before (black) and after (blue) synthesis of MoS_2/WSe_2/sapphire compared to pure MoS_2 (red), where the locations are indicated in Supplementary Figure 5a. From the Raman spectra, we find that the WSe_2 is replaced by sulfur and molybdenum to form WS_2 and MoS_2. The PL of WSe_2 is dramatically reduced and a signature of MoS_2 is found in the inner flake regime, which further suggests that the WSe_2 has been damaged and/or replaced during CVD process. The replacement of WSe_2 by sulfur and molybdenum on sapphire substrates may begin at defect sites within the flake or the chosen conditions for synthesis TMDs on graphene and sapphire, which needs further investigation.

Supplementary Note 5 Scanning Tunneling Spectroscopy: Error estimations for Band gap, VBM and CBM.

In order to determine the electronic band gap value obtained for ML WSe_2 and ML MoS_2/ML WSe_2 the dI/dV curves in the STS were differentiated from an average of at least ten I-V curves acquired sequentially at a fixed position. The data were acquired close to the center of the heterostructure domains in order to avoid probing edge states along TMD edges which overlap with graphene.[12] The bandgap, the CBM and the VBM value and error given in the main text were estimated from several measurements. The CBM and VBM were acquired from the points where fitting lines intersect the both ends of bandgap region (where dI/dV curves present a parallel line). The bandgap is then determined by: [$CBM_{average} - VBM_{average}$]. As an example, Supplementary Figure 6 shows the WSe_2 bandgap value extrapolated from 6 different dI/dV spectra. The mean bandgap is estimated at 1.83 eV with a standard deviation equal to 0.07, i.e. $E_g(WSe_2) = 1.83 \pm 0.07$ eV.

Supplementary Note 6 Additional I-V plots from the CAFM measurement.

The measured spots were confirmed as the double junctions in the topography/CAFM images before the I-V curve measurement was accomplished. The AFM tip-loading force was increasing up to 5 nN, after which the curves are virtually identical due to the optimized contact area between tip and the samples as well as the removal of possible air/water layer in between.[13] Each plot in Supplementary Figure 7 is after the average of 3 repeated measurements on the same spot. The corresponding peak-to-valley current ratio (PVCR) and the voltage of the peak maximum are labeled. The variation in the peak position and width is likely related to defect formation within the layers and the interface of the layers.

Supplementary Note 7 Negative Differential Resistance in 2D heterostructures.

Resonant tunneling between two spatially separated quantum states can be used to realize negative differential conductance. Negative differential conductance holds the key for novel nano-electronic design options utilizing bistability and positive feedback. Novel memories, multi-valued logic and inductor-free compact oscillators and other electronic applications can benefit from a low power, low voltage negative differential conductance device. The resonant tunneling diode (RTD) has been a subject of intense study and design optimization, in Silicon Germanium and III-V heterostructure material systems for many years now. While theoretically capable of operating in extremely narrow voltage windows, the negative differential conductance of a RTD, particularly at room temperature, is limited by scattering mechanisms, related to interfacial imperfections, which are unavoidable even when utilizing high vacuum advanced epitaxial growth technique. The interface related scattering reduces the sensitivity of resonant tunneling to an external bias, thereby increasing the voltage window over which negative differential conductance regime is observed. van der Waals epitaxy of 2D materials can mitigate these issues and provide a materials platform for device engineers to obtain energetically sharp NDR features at room temperature leading to novel low power quantum tunneling devices. Absence of dangling bond in the interface and reduced interface roughness scattering in these two dimensional materials based devices makes it possible to obtain sharp NDR with very low full width half max voltage and relatively high PVCR in room temperature (Supplementary Table 2).

Supplementary References:

1. Chiu, M.-H. *et al.* Spectroscopic signatures for interlayer coupling in MoS_2-WSe_2 van der Waals stacking. *ACS Nano* **8,** 9649–9656 (2014).

2. Fang, H. *et al.* Strong interlayer coupling in van der Waals heterostructures built from single-layer chalcogenides. *Proc. Natl. Acad. Sci. U. S. A.* **111,** 6198–202 (2014).

3. Coy Diaz, H., Addou, R. & Batzill, M. Interface properties of CVD grown graphene transferred onto MoS_2(0001). *Nanoscale* **6,** 1071–2078 (2014).

4. McDonnell, S., Addou, R., Buie, C., Wallace, R. M. & Hinkle, C. L. Defect-dominated doping and contact resistance in MoS_2. *ACS Nano* **8,** 2880–2888 (2014).

5. McDonnell, S. *et al.* Hole Contacts on Transition Metal Dichalcogenides: Interface Chemistry and Band Alignments. *ACS Nano* **8,** 6265–6272 (2014).

6. Mishchenko, A. *et al.* Twist-controlled resonant tunnelling in graphene/boron nitride/graphene heterostructures. *Nat. Nanotechnol.* **9,** 808–813 (2014).

7. Nguyen, L.-N. *et al.* Resonant tunneling through discrete quantum states in stacked atomic-layered MoS_2. *Nano Lett.* **14,** 2381–2386 (2014).

8. Jin, N. *et al.* Diffusion Barrier Cladding in Si / SiGe Resonant Interband Tunneling Diodes and Their Patterned Growth on PMOS Source / Drain Regions. *IEEE Trans. Electron Devices* **50,** 1876–1884 (2003).

9. Huang, J.-K. *et al.* Large-area synthesis of highly crystalline WSe_2 monolayers and device applications. *ACS Nano* **8,** 923–930 (2014).

10. Lin, Y.-C. *et al.* Direct Synthesis of Van der Waals Solids. *ACS Nano* **8,** 3715–3723 (2014).

11. Wang, H. *et al.* $MoSe_2$ and WSe_2 nanofilms with vertically aligned molecular layers on curved and rough surfaces. *Nano Lett.* **13,** 3426–3433 (2013).

12. Zhang, C., Johnson, A., Hsu, C.-L., Li, L.-J. & Shih, C.-K. Direct imaging of band profile in single layer MoS2 on graphite: quasiparticle energy gap, metallic edge states, and edge band bending. *Nano Lett.* **14,** 2443–2447 (2014).

13. Lee, G.-H. *et al.* Electron tunneling through atomically flat and ultrathin hexagonal boron nitride. *Appl. Phys. Lett.* **99,** 243114 (2011).
